# JQ1 attenuates contrast-induced acute kidney injury through the upregulation of autophagy and inhibition of inflammation

**DOI:** 10.1007/s11255-023-03718-7

**Published:** 2023-08-07

**Authors:** Linghong Ge, Juntao Chen, Xueying Ren, Chunqi Huang, Danqing Dong, Zhou Yin

**Affiliations:** 1 Department of Laboratory Medicine, The Second Affiliated Hospital of Zhejiang Chinese Medical University, Hangzhou, Zhejiang China; 2 Department of Urology, Zhongshan Hospital, Fudan University, Shanghai, China

**Keywords:** Contrast media, Acute kidney injury, JQ1, Autophagy, Inflammation

## Abstract

**Purpose:**

Contrast-induced acute kidney injury (CI-AKI) is the third most common cause of hospital-acquired AKI. However, there is a paucity of efficacious interventions for the management of CI-AKI. Here, we aim to investigate the effects of JQ1 in CI-AKI and provide theoretical data and a  foundation for  novel ideas for the clinical treatment of CI-AKI.

**Methods:**

In this study, we performed in vivo and in vitro experiments with mice and HK2 cells injury models respectively. The levels of serum creatinine (Cr) and blood urea nitrogen (BUN) were determined by an automatic analyzer for the measurements of renal function. The viability of HK-2 cells was analyzed using the Cell Counting Kit-8 (CCK-8) kit. Additionally, the kidney changes in the mice were detected using histopathology (H&E) and immunofluorescent staining. The mRNA and protein expressions were assessed using Quantitative real-time PCR and western blot, respectively. Autophagy and apoptosis was analyzed by Transmission electron microscopy (TEM) and TUNEL assay respectively.

**Results:**

The results demonstrated that JQ1 exhibited potency of attenuating CI-AKI in mouse and HK2 cells. JQ1 increased the expression levels of Atg5, Atg7 and LC3B-II, and decreased the protein levels of p62 in the kidney and HK-2 cells. However, the combined use of JQ1 with chloroquine reversed the effects of JQ1. JQ1 also inhibited the inflammatory cells and downregulated the expression of some inflammatory cytokines (IL-6, IL-1β, TNF-α, and IFN-γ).

**Conclusion:**

JQ1 protects against CI-AKI by promoting autophagy and inhibiting inflammation and JQ1 may be a promising therapeutic strategy for CI-AKI.

## Introduction

Contrast-induced acute kidney injury (CI-AKI) is a major health problem that occurs in approximately 30% of patients who receive iodinated contrast media. CI-AKI is the third most common form of hospital-acquired AKI [1]. More than 2 million heart catheterizations are performed [2] and more than 30 million doses of contrast medium are used every year [3]. Previous studies have suggested that pretreatment with antioxidants and optimization of hydration protocols decrease nephrotoxic effects [4–6]. However, a recent study has demonstrated the absence of benefits with routine antioxidant and sodium bicarbonate therapies for the prevention of CI-AKI and death [7]. The mechanisms of CI-AKI have still not been elucidated completely. The relative factors of CI-AKI involve the direct nephrotoxic effects of iodinated contrast media, apoptosis, immune/inflammation, oxidative stress, hemodynamic changes, and epigenetic regulation [8]. Thus, exploration of the underlying mechanisms of CI-AKI and investigation of new drugs will aid in the development of novel preventive and therapeutic strategies.

JQ1, a first-in-class potent and selective inhibitor of Bromodomain-containing protein 4 (BRD4), displaces Bromodomain and Extraterminal (BET) bromodomains from chromatin and interferes with BRD4 function [9]. JQ1 is widely used for studying tumor biology, inflammation, and other research [10]. Moreover, recent studies suggest that JQ1 can activate autophagy in many disease models. Zou et al. reported that JQ1 can activate PINK1/Parkin-mediated mitophagy and prevent cardiomyopathy in high-fat diet diabetic mice [11]. Another study reported that JQ1 suppresses bladder cancer cell proliferation by inducing autophagy [12]. Additionally, studies indicated that JQ1 can inhibit the expression of inflammatory cytokines. A study by Maksylewicz et al. on periodontitis indicated that JQ1 can inhibit the expression of inflammatory cytokines and chemokines [13]. Moreover, another study noted that JQ1 attenuated gastric inflammation and immune cell infiltration in mice infected with Helicobacter pylori [14]. Thus, these studies indicated that JQ1 has immunosuppressive potential in chronic infections caused by some pathogenic bacteria and can promote autophagy and inflammation.

Autophagy stabilizes renal cell survival and kidney function by contributing to the maintenance of mitochondrial homeostasis via degradation of damaged mitochondria [15]. During AKI, activation of autophagy can play a protective role in kidney injury, and a deficiency of autophagy makes the kidneys vulnerable to ischemic injury and renal toxicity [16]. A study has indicated that upregulation of Beclin1 and increased autophagy may restore cell vitality and suppress apoptosis in HK2 cells during contrast media treatment [17]. Additionally, an excessive inflammatory response is responsible for high mortality in patients with CI-AKI by severe damage to the tubules and epithelial cell death [18]. Lin et al. reported that upregulation of HIF1A and BNIP3-mediated mitophagy inhibit NLRP3 inflammasome and attenuate apoptosis in CI-AKI [19].

Previous studies have demonstrated the potential of JQ1 in the treatment of CI-AKI. Therefore, we investigated whether JQ1 treatment could confer protection from iohexol injury in a CI-AKI mice model. This preliminary exploration is aimed at providing theoretical data and a basis for new ideas for the clinical treatment of CI-AKI.

## Materials and methods

### Mice

All 6–8 weeks old male C57BL/6 mice were purchased from SLAC Laboratory Animal Center and housed under specific pathogen-free conditions. All animal experiments were performed according to the guidelines of the Care and Use of Laboratory Animals of the Laboratory Animal Ethical Commission of Fudan University and were approved by the Animal Ethical Committee of Zhongshan Hospital, Fudan University, Shanghai, China. All animal experiments were complied with the ARRIVE guidelines and conducted according to the National Institutes of Health Guide for the Care and Use of Laboratory Animals.

### CI-AKI in mice

Mice were anesthetized with 1% pentobarbital and their right kidneys were excised. After 3 weeks, mice underwent water deprivation for 2 days and then were administrated with iohexol (GE Healthcare, Omnipaque 350, USA) 10 mg/kg via tail vein. JQ1 (50 mg/kg, Selleck, USA) was administered by intraperitoneal injection at day -2 and day 0. Chloroquine (CQ) (50 mg/kg, Selleck, USA) was administered by intraperitoneal injection during iohexol injection. Then the mice were sacrificed at 24 h post iohexol administration and the kidney and serum were harvested for further analysis.

### HK2 cells culture

Human renal tubular epithelial cells (HK-2) were obtained from the Shanghai cell bank of the Chines Academy of Sciences. The HK-2 cells were cultured in 1:1 DMEM/F-12 (ThermoFisher Scientific, USA) with 10% fetal bovine serum and 1% penicillin/streptomycin solution in 5% CO_2_ humidified air at 37 °C. The medium was replaced every 2–3 days. The HK2 cells were randomly divided into four groups: (1) control: the cells were only treated with normal medium; (2) JQ1 group: the cells were treated with 250 nM JQ1 for 4 h; (3) Iohexol group: the cells were treated with iohexol (100 mgI/ml) for 4 h; (4) JQ1 + Iohexol group: the cells were treated with 250 nM JQ1 and iohexol (100 mgI/ml) together for 4 h. Then the cells were collected for cell viability analysis and apoptosis analysis.

### Serum measurements

The levels of serum creatinine (Cr) and blood urea nitrogen (BUN) were determined by an automatic analyzer (Roche, Basel, Switzerland) for the measurements of renal function.

### Histology and histopathology (H&E) and immunofluorescent staining

The kidney tissues were fixed in 4% paraformaldehyde and then embedded in paraffin. Then 5 μm thick sections were cut for H&E staining. The tubular injury score was evaluated according to the following grades: grade 0, normal; grade 1, < 25%; grade 2, 25–49%; grade 3, 50–74%; grade 4, ≥ 75%. Immunofluorescent staining was performed according to the manufacturer’s instructions. The antibodies specific for F4/80 (Abcam, ab111101) and Ly6G (Abcam, ab238132) were incubated at 4 °C overnight, and the secondary antibodies were incubated for 1 h at room temperature. A fluorescence microscope was used to observe.

### Quantitative real-time PCR

Gene expression of IL-1β, TNF-α, INF-γ, and IL-6 was assessed by quantitative real-time PCR. Kidney total RNA was extracted using TRIzol reagent (Thermo Scientific, USA) according to the manufacturer’s instructions. 1 μg of total RNA was reverse-transcribed to cDNA using Synthesis Kit (Vazyme, Nanjing, China). Real-time PCR reactions were performed using SYBR qPCR Master Mix (Vazyme, Nanjing, China). The data were analyzed using the 2^−ΔΔ^Ct method. The primers for the genes of interest were as follows (5′–3′): IFN-γ (TCA AGT GGC ATA GAT GTG GAA GAA and TGG CTC TTG CAG GAT TTT CAT G), IL-6 (CCA GTT TGG TAG CAT CCA TCA T and GAG AAA GAG TTG TGC AAT GGC), TNF-α (CCC TCA CAC TCA GAT CAT CTT CT and GCT ACG ACG TGG GCT ACA G), IL-1β (GCA ACT GTT CCT GAA CTC AAC T and ATC TTT TGG GGT CCG TCA ACT).

### Transmission electron microscopy (TEM)

The fresh renal cortex (1 mm^3^) was harvested and prefixed with 2.5% glutaraldehyde in PBS for 24 h at 4 °C. After being fixed in 1% OsO4 for 1 h, samples were washed with double distilled water three times and stained in 2% uranyl acetate, dehydrated in a graded alcohol series, and embedded in Epon 812 (SPI, 660-AB), sectioned, and stained with lead citrate. Finally, the sections were detected with a TEM (Philips, Netherlands).

### Western blot

For immunoblotting, cells and tissues were lysed with RIPA buffer. Protein concentrations were determined using a BCA Protein Assay Kit (ThermoFisher, USA). Then proteins were boiled, separated on SDS-PAGE gel electrophoresis, and transferred onto polyvinylidene fluoride membranes (ThermoFisher, USA). Membranes were blocked with 5% bovine serum albumin in TBST (Sigma-Aldrich) for 1 h and incubated overnight at 4 °C with primary antibodies (p62, LC3B, ATG5, ATG7, and β-actin, all from Cell Signaling Technology, USA). Afterward, the membranes were incubated with the secondary antibody. Finally, protein blots were developed by the Molecular Imager ChemiDoc XRS + System.

### Cell viability analysis

The viability of HK-2 cells was analyzed using the Cell Counting Kit-8 (CCK-8) kit (Beyotime Biotechnology, China). The HK-2 cells were cultured in a 96-well plate and treated with JQ1 or/and iohexol. Then the cells were incubated with 10 μL of CCK-8 solution for 2 h. The absorbance of the mixture was measured at 450 nm using BioTak CytationTM3.

### Apoptosis analysis

Apoptosis was analyzed by TUNEL assay, and flow cytometry. TUNEL staining (Beyotime Biotechnology, China) was performed following the manufacturer’s instructions. For Annexin V-FITC staining (Beyotime Biotechnology, China), the cells were suspended in 100 μL of Binding Buffer and then were treated with 5 μL Annexin V-FITC and 5 μL of PI in the dark for 15 min. Finally, they were analyzed by flow cytometry.

### Statistics

Statistical analysis was performed using Prism 7.0.4 (GraphPad Software, USA). Qualitative data were analyzed by two-tailed unpaired Student's t test between two groups and expressed as means ± standard error of the mean (SEM). P < 0.05 was considered significant.

## Results

### JQ1 treatment attenuates CI-AKI

**Fig. 1:**
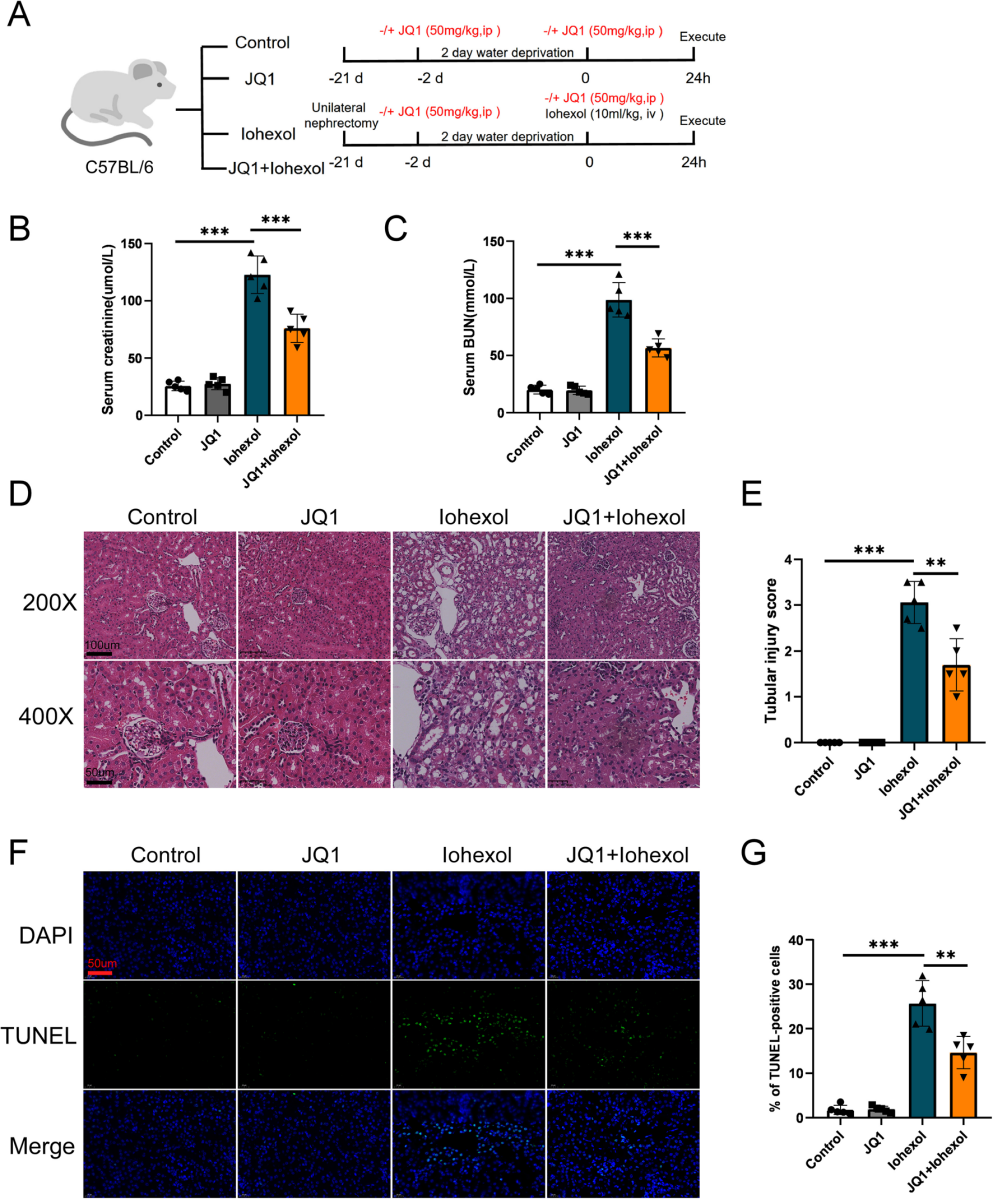
JQ1 administration ameliorated renal function and reduced renal tubular injury score, and cell apoptosis rate after iohexol-treatment in vivo. **A** Diagrammatic representation of four groups of mice (Control, JQ1, Iohexol, and JQ1 + Iohexol). **B** The level of Serum creatinine in different groups. (C) The level of Serum BUN in different groups. **D**, **E** Representative histology (200× and 400×) and pathological tubular injury score in the renal cortex by H-E staining. **F**, **G** Apoptosis was evaluated by TUNEL staining (200×) and quantification of TUNEL-positive cells. (*P < 0.05, **P < 0.01 and ***P < 0.001)

To investigate the effect of JQ1 on CI-AKI, we used four groups of CI-AKI mice (Fig. 1A). First, levels of Cr and BUN were measured 24 h after iohexol injection. The Cr and BUN levels in the iohexol group were significantly higher than those in control group (Fig. 1B, C); this validated the success of our model. Further, the Cr and BUN levels in the JQ1 + iohexol group were significantly lower than those in iohexol group (Fig. 1B, C). The H&E staining of the kidney sections showed the cytoplasmic vacuolar changes, cast formation, loss of brush border, and interstitial inflammation in the iohexol group, that were alleviated by administration of JQ1 (Fig. 1D, E). Additionally, the TUNEL assay showed that the number of apoptotic renal tubular epithelial cells (TECs) in the JQ1 + iohexol group was lower than that in iohexol group (Fig. 1F, G). These findings suggest that JQ1 attenuates the iohexol-induced renal injury.

### JQ1 protects the HK2 cells against iohexol-induced injury in vitro

**Fig. 2:**
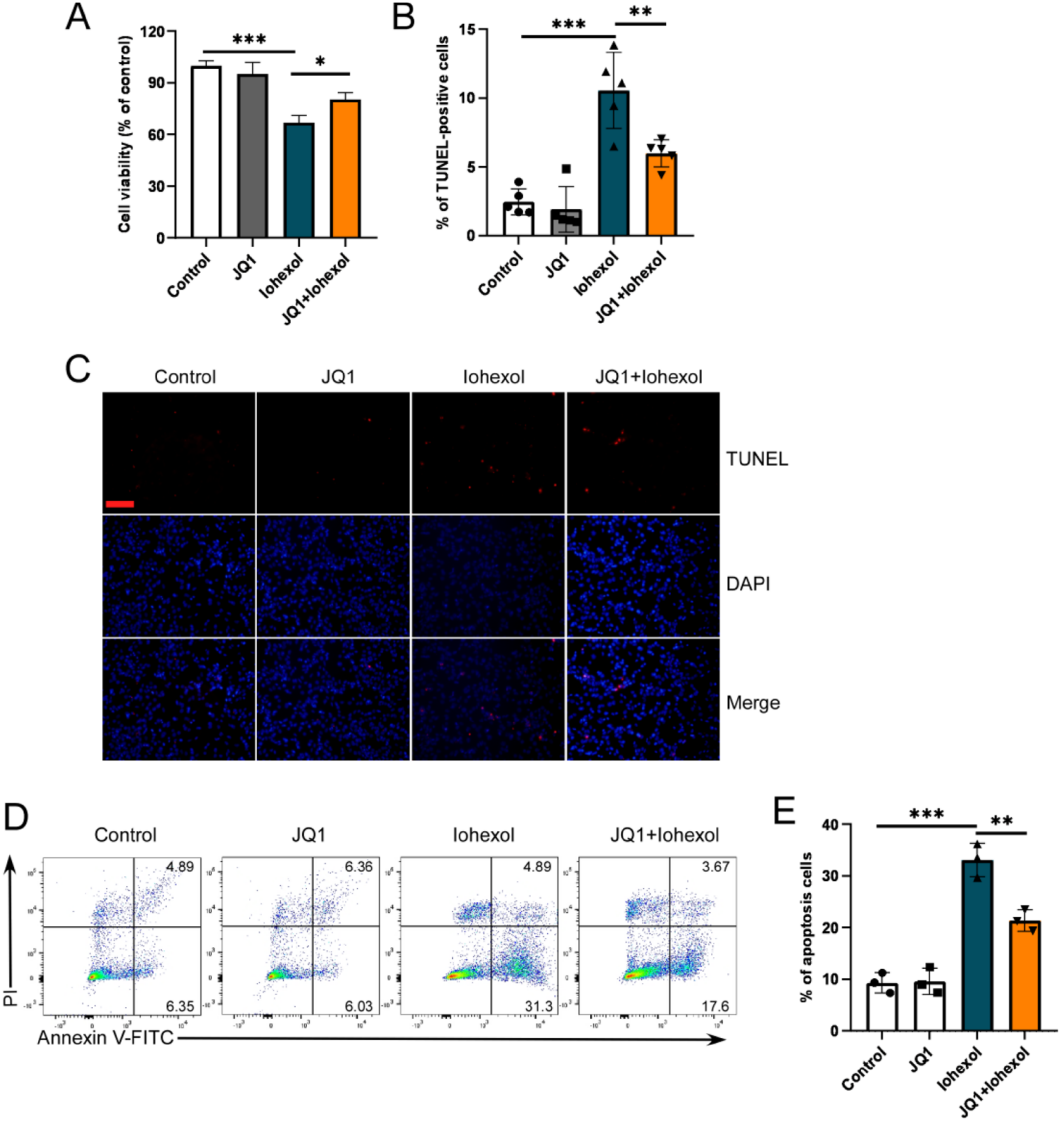
JQ1 protected the HK2 cells against iohexol induced injury in vitro. **A** Cell viability of different treated HK-2 cells was evaluated by CCK-8. **B**, **C** Apoptosis was evaluated by TUNEL staining (200× , scale bar: 100 μm) and quantification of TUNEL-positive cells. (D and E) Representative images and quantification of cell apoptosis by flow cytometry. (*P < 0.05, **P < 0.01, and ***P < 0.001)

To examine the effect of JQ1 on iohexol-induced HK2 cells in vitro, we cultured HK2 cells with iohexol and/or JQ1 and performed cell viability and cell apoptosis assays. The analyses indicated that iohexol treatment decreased cell viability and increased cell apoptosis (Fig. 2A–E). Further, the CCK-8 assay suggested that JQ1 + iohexol treatment recovered the cell viability of HK2 cells more than that in iohexol group (Fig. 2A). The PI–Annexin V and TUNEL assays revealed that JQ1 treatment alleviated cell apoptosis (Fig. 2B–E). Taken together, these findings suggested that JQ1 alleviated the iohexol-induced injury of HK2 cells in vitro.

### JQ1 promotes autophagy in the renal tubules, in vivo and in vitro

**Fig. 3:**
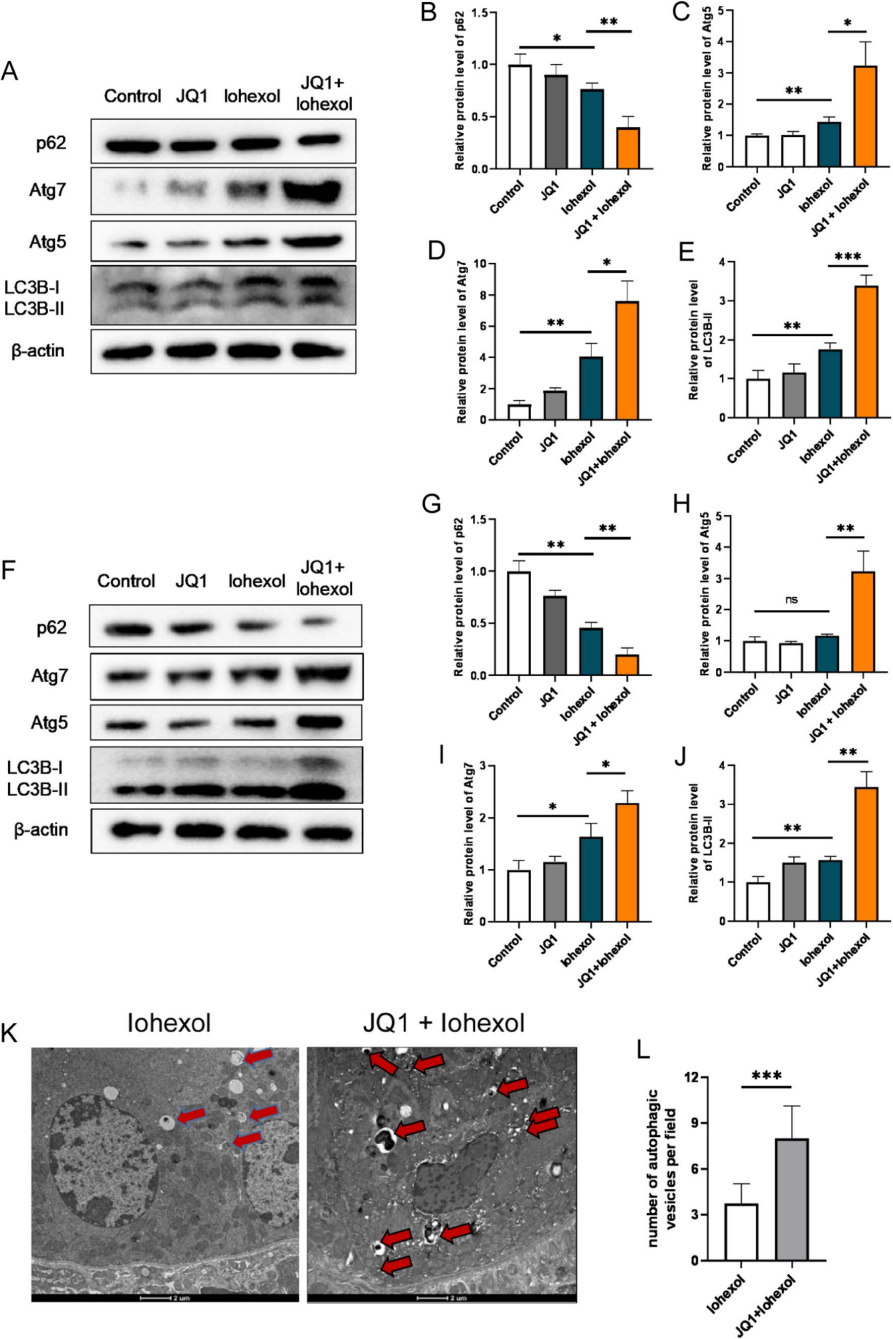
JQ1 promoted autophagy in the renal tubular both in vivo and in vitro. **A**–**E** Immunoblotting analysis and quantification of p62, Atg5, Atg7, and LC3B-II of different groups of kidney tissue in vivo. **F**–**J** Immunoblotting analysis and quantification of p62, Atg5, Atg7, and LC3B-II of different groups of HK2 cells in vitro. **K**, **L** Representative TEM images and quantification of autophagic vesicles in renal TECs of Iohexol and JQ1 + Iohexol groups of mice. Autophagic vesicles were indicated by arrows (*P < 0.05, **P < 0.01, and ***P < 0.001)

As autophagy plays an important role in CI-AKI, we evaluated that whether levels of autophagy-related proteins change after iohexol treatment. Western blotting analysis showed that protein levels of Atg5, Atg7, and LC3BII were significantly increased and protein levels of p62 were significantly decreased in the iohexol group than those in control group (Fig. 3A–E). Moreover, JQ1 treatment further increased protein levels of Atg5, Atg7, and LC3BII and decreased protein levels of p62 than those in iohexol group (Fig. 3A–E). Thus, the results indicate that JQ1 promotes autophagy in CI-AKI mice. Next, we evaluated the autophagy levels in HK2 cells in vitro, which suggested a similar outcome (Fig. 3F–J). Additionally, TEM is another important method to observe autophagy. Therefore, we observed autophagosomes in renal tissues; the analyses suggested that the number of autophagic vesicles in the JQ1 + iohexol group was significantly higher than that in iohexol group (Fig. 3K, L).

### CQ reduces the protective effects of JQ1 by blocking autophagy

**Fig. 4:**
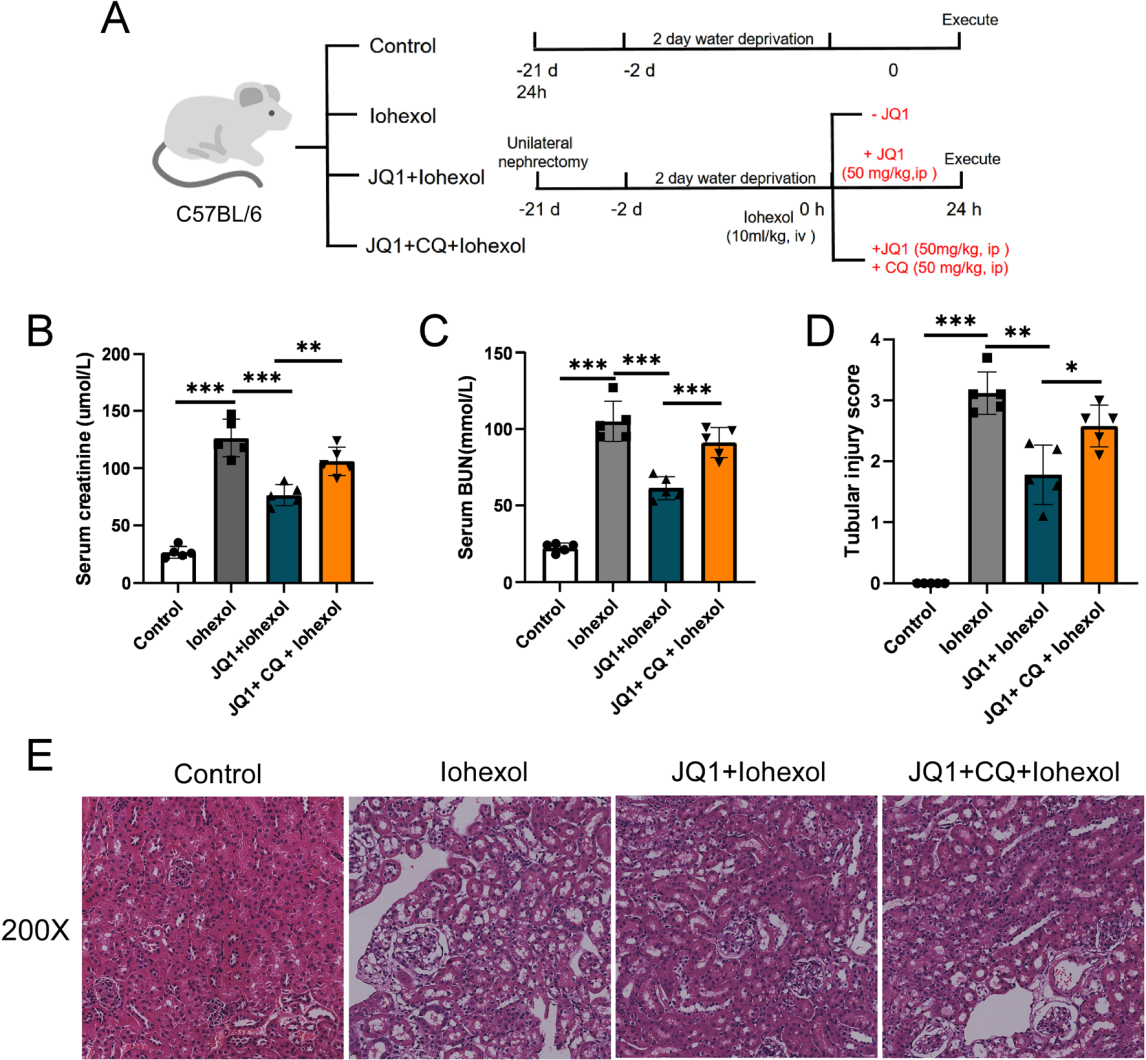
CQ weakened the protective effect of JQ1 by blocking autophagy. **A** Diagrammatic representation of four groups of mice (Control, Iohexol, JQ1 + Iohexol, and JQ1 + CQ + Iohexol). **B** The level of Serum creatinine in different groups. **C** The level of Serum BUN in different groups. **D**, **E** Representative histology (200×) and pathological tubular injury score in the renal cortex by H-E staining. (*P < 0.05, **P < 0.01 and ***P < 0.001)

To verify that the protective effects of JQ1 were mediated through increased autophagy signaling, we utilized CQ, an inhibitor of autophagy, and divided mice into four groups (Fig. 4A). The Cr and BUN levels were measured; the results showed that a combined usage of CQ and JQ1 significantly increased the Cr and BUN levels in the JQ1 + CQ + iohexol group than those in JQ1 + iohexol group (Fig. 4B, C). Additionally, the HE staining showed that pathological injury was more severe and the tubular injury score was higher in the JQ1 + CQ + iohexol group than that in JQ1 + iohexol group (Fig. 4D, E). However, the Cr and BUN levels in the JQ1 + CQ + iohexol group were still lower and the pathological injury was milder than those in iohexol group. These findings indicate that the blockade of autophagy by CQ could nearly abolish the protective effects of JQ1 on CI-AKI.

### JQ1 exerts a protective effect against inflammation induced by iohexol

**Fig. 5:**
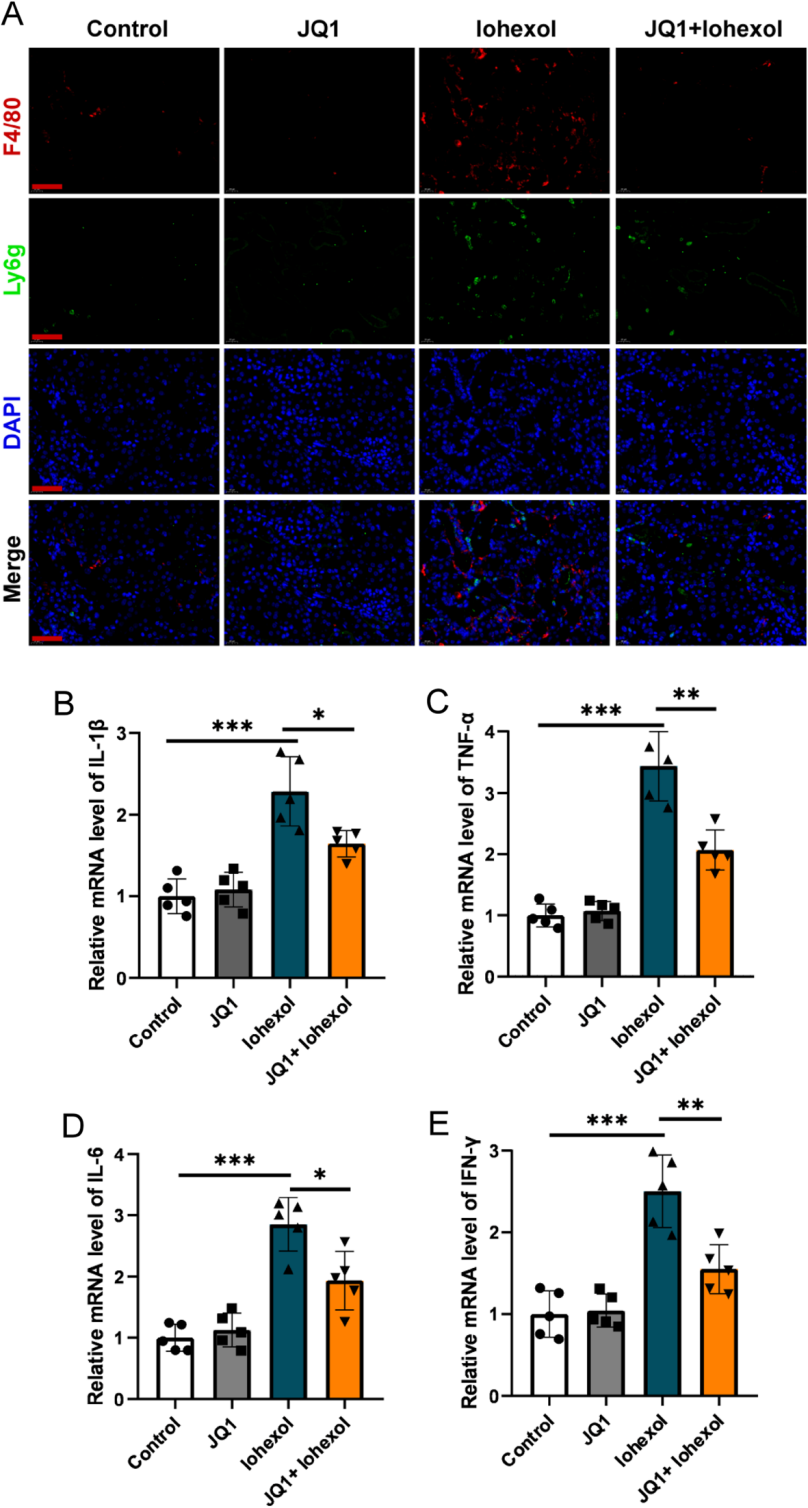
JQ1 exerted a protective effect against inflammation induced by iohexol. (A) Immunofluorescence images of F4/80 (red) and Ly6g (green) in Control, JQ1, Iohexol, and JQ1 + Iohexol groups (400× , scale bar: 50 μm). **B**–**E** mRNA level of IL-1β, TNF-α, IL-6, IFN-γ in Control, JQ1, Iohexol, and JQ1 + Iohexol groups. (Data are representative of three independent experiments. *P < 0.05, **P < 0.01, and ***P < 0.001)

To elucidate the extent of protection by JQ1 on inflammation, the number of inflammatory cells such as neutrophil and macrophage, and levels of inflammatory cytokines, such as IL-1β, TNF-α, IFN-γ, and IL-6 were measured. The immunofluorescent staining showed that the number of neutrophils and macrophages were less in the JQ1 + iohexol group than that in iohexol group (Fig. 5A). Correspondingly, the mRNA levels of these inflammatory cytokines were lower in the JQ1 + iohexol group than those in iohexol group (Fig. 5B–E). Therefore, JQ1 may protect the kidney against iohexol-induced injury via an anti-inflammatory effect.

## Discussion

Iodinated contrast media has become pivotal in clinical practice with the advent of interventional radiologic technology [18]. However, contrast material exposure may increase the risk of AKI. The iodinated contrast-induced AKI is the third most frequent cause of high morbidity and mortality, and accounts for 11% of all cases of AKI in hospitalized patients [20, 21]. CI-AKI may prolong hospital stays and also accelerate the progression of chronic kidney disease [20]. Therefore, finding novel and effective drugs to prevent and treat CI-AKI is necessary.

JQ1 is widely studied in different fields, such as tumor biology, inflammation, viral infection, and neurological disorders [10, 22]. In this study, we investigated the effect of JQ1 on CI-AKI. The analyses suggested that CI-AKI mice had significant renal injury based on both serum Cr levels and pathological damage of the renal tubular cells. However, JQ1 treatment significantly improved kidney function (decreased the Cr levels and rescued damaged pathology) than CI-AKI without JQ1 treatment. Furthermore, we tested the effects of JQ1 in vitro and found that it increased cell viability and decreased cell apoptosis of HK2 cells upon iohexol treatment. To the best of our knowledge, this is the first study to report that JQ1 exerts protective effects on CI-AKI.

The mechanisms underlying CI-AKI are complex and have not been fully elucidated. As described in previous studies, the main pathogenesis includes direct toxicity of contrast media to TECs, ischemia, and hypoxia; increased production of ROS and induced oxidative stress; epigenetic regulation; apoptosis; and inflammatory reactions [1, 8, 23]. Several recent studies have described the relationship between autophagy and CI-AKI. Ni et al. reported that mitophagy confers protection against CI-AKI [24] and that the upregulation of HIF1A and BNIP3-mediated mitophagy via inhibition of NLRP3 inflammasome may also attenuate apoptosis in CI-AKI [19]. Another study reported that regulation of mitophagy and senescence by paricalcitol may attenuate CI-AKI [25]. JQ1 has been validated as an activator of autophagy in previous studies [11, 12, 26]. This study showed that JQ1 treatment could upregulate the expression of autophagy-related proteins (ATG5, ATG7, Beclin1, and LC3BII/LCB3I) and decrease expression of p62 in CI-AKI mice. Moreover, we observed an increased number of autophagosomes in the JQ1 + CI-AKI group than in control group. Next, in vivo inhibition of the autophagy pathway by CQ further confirmed that JQ1 partially attenuated CI-AKI by increasing the autophagy pathway.

Increased inflammation is another important mechanism of CI-AKI. Previous studies have demonstrated that contrast treatment in experimental mice increased the levels of inflammatory cytokines, including IL-6 and TNF-α [27, 28]. In contrast, the renal injury could be significantly alleviated upon inhibition of inflammation [27, 28]. Interestingly, a recent study showed that NLRP3 is activated by several stimuli, including osmotic stress and ROS induced directly by contrast, along with release of damage-associated molecular patterns from injured TECs [8, 29]. JQ1 has been validated as an inflammation inhibitor in many experimental models, such as renal cell carcinoma, central nervous system, Alzheimer's disease model, and pulmonary arterial hypertension [30–33]. In this study, using the CI-AKI mice model, we detected changes in several inflammatory cytokines. Furthermore, JQ1 treatment of mice significantly downregulated the mRNA expression of proinflammatory cytokines (IL-1β, TNF-α, INF-γ, and IL-6) than expression in mice without JQ1 treatment. Therefore, the protective effect of JQ1 on the CI-AKI mice may be via activation of autophagy and also with inhibition of inflammation. However, the underlying mechanism by which JQ1 inhibits inflammation, and whether the number of inflammation-related cells, such as macrophages and neutrophils changes, need to be further investigated.

A previous study has showed that JQ1 abrogated experimental renal inflammation in the unilateral ureteral obstruction mice model [34] and alleviated apoptosis induced by renal ischemia/reperfusion injury [35]. In our CI-AKI model, that also included the pathology of apoptosis and inflammation, JQ1 increased autophagy in both mice and HK2 cells treated with iohexol and anti-inflammatory effects in mice treated with iohexol. Moreover, autophagy inhibition by CQ reversed the beneficial effects of JQ1, thus confirming that JQ1 protects the kidney from CI-AKI via activation of autophagy. However, the detailed molecular mechanism by which JQ1 increases levels of autophagy-related proteins and inhibits inflammation remains unknown. Additionally, the relationship between autophagy and inflammation in systems treated with JQ1 and iohexol also remains unclear. Therefore, future studies focused on these aspects will aid in better understanding the pathogenesis of CI-AKI and elucidating the therapeutic potential of JQ1.

## Conclusions

**Fig. 6:**
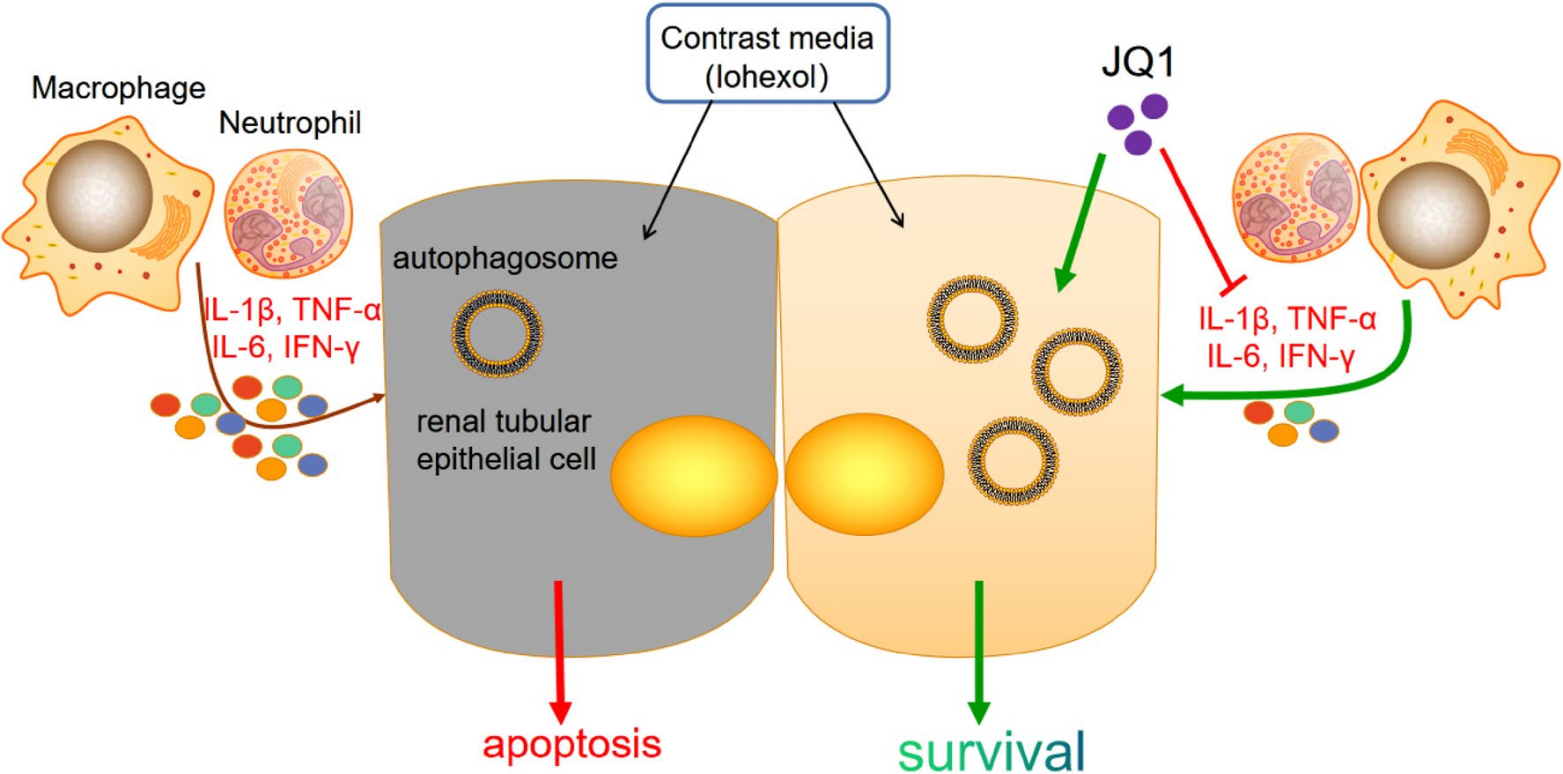
The model of the experiment: JQ1 protect renal TECs survival from Iohexol injury by activating the autophagy signaling pathway and inhibiting inflammatory cytokines such as IL-6, IFN-γ, IL-1β, and TNF-α

This study demonstrated that JQ1 confers kidney protection in a CI-AKI model via activation of autophagy signaling pathway and anti-inflammation activity (Fig. 6). The findings may aid in better understanding the mechanisms of CI-AKI and finding therapeutic interventions to attenuate CI-AKI in patients.
